# Retraction Note: Post-operative blood pressure and 3-year major adverse cardiac events in chinese patients undergoing PCI

**DOI:** 10.1186/s12872-023-03457-8

**Published:** 2023-08-24

**Authors:** Lijun Gan, Dandan Sun, Yuntao Cheng, Deyang Wang, Fen Wang, Lin Wang, Wei Li, Dandan Shen, Daotong Guo, Zonglei Zhang, Haiyan Wang, Jinli Li, Yong Yang, Tao Liang

**Affiliations:** 1 Department of Cardiology, Affiliated Hospital of Jining Medical University, Jining, Shandong China; 2 Cardiac Emergency Department, Affiliated Hospital of Jining Medical University, Jining, Shandong China; 3 Catheterization Room, Affiliated Hospital of Jining Medical University, Jining, Shandong China; 4 Department of Nursing, Affiliated Hospital of Jining Medical University, Jining, Shandong China; 5 School of Nursing, Peking Union Medical College, Beijing, China

**Retraction Note: BMC Cardiovascular Disorders (2021) 21:623**

10.1186/s12872-021-02435-2

The Editor has retracted this article at the corresponding author’s request because after the publication of the article, the authors have found that the conclusion of this article cannot be validated by a larger sample-size study from the same cohort. None of the authors responded to any correspondence from the editor/publisher about this retraction.

